# Trends and Prevalence of Surgical Methods in Umbilical Hernia Repairs in Sweden: A Nationwide Population-Based Registry Cohort Study

**DOI:** 10.3389/jaws.2026.15685

**Published:** 2026-01-26

**Authors:** Mathias Bergström, Björn Widhe, Sven Bringman, Maria Melkemichel

**Affiliations:** 1 Department of Clinical Science and Education, Södersjukhuset, Karolinska Institutet, Solna, Sweden; 2 Department of Surgery, Södertälje Hospital, Södertälje, Sweden; 3 Department of Breast, Endocrine Tumors and Sarcoma, Karolinska University Hospital, Solna, Sweden

**Keywords:** mesh repair, prevalence, suture repair, trends, umbilical hernias

## Abstract

**Background:**

Umbilical hernia repairs (UHRs) are commonly performed worldwide, yet knowledge regarding methods of repair remains limited. This study aimed to assess the trends and prevalence of suture versus mesh repairs for UHRs in Sweden over time.

**Methods:**

This observational population-based registry study utilised prospectively collected data from the nationwide Swedish Perioperative Registry. Patients aged ≥18 who received a UHR between the years 2017–2022 were eligible. Surgical units were categorised into six healthcare regions. The primary outcome was to observe the trend in repair methods (suture vs. mesh) over time. The secondary outcome included descriptive patient- and hernia characteristics of the UHRs, along with regional variations.

**Results:**

Out of 10,374 primary elective UHRs, mesh was used in 47.9% of cases, with 14.2% performed laparoscopically. Mesh repairs were less common in women (38.7%) compared to men (52.1%) (p < 0.001). Suture repair patients had a lower median age (49 years) and BMI (27.2 kg/m^2^) compared to those with mesh repairs (55 years, BMI 29.7 kg/m^2^) (p < 0.001). A higher ASA class (3–4) was more common for mesh repair recipients (17.1%) compared to suture repair recipients (10.9%). The use of mesh repairs increased from 46.2% to 49.4% over the study period (p = 0.063), with only the Southern healthcare region showing a significant rise from 25.0% to 56.1% (p < 0.001).

**Conclusion:**

The use of mesh repairs has not yet significantly influenced UHR practices in Sweden. Mesh was used more frequently among men, obese patients, older individuals, and those with greater co-morbidities.

## Introduction

An umbilical hernia repair (UHR) is a widespread surgical procedure. It is the most frequently repaired hernia after inguinal hernias [1]. Despite its high prevalence, UHR has not been studied as extensively as other common surgical conditions.

In recent years, numerous studies, including randomised controlled trials [2–4], cohort studies [5–10], and meta-analyses [11–13], have compared suture repair to various types of mesh repair, significantly advancing the understanding of UHR techniques. Mesh repair for umbilical hernias (UH) has consistently shown a reduction in recurrence rates [3, 5, 14, 15]. However, findings are conflicting regarding the potential increase in surgical site occurrences associated with mesh repairs [12, 13, 16]. These discrepancies likely derive from variations in mesh techniques and hernia defect sizes, underscoring the need for a tailored approach in each case. Concerns regarding a potentially increased risk of complications with mesh use may also contribute to hesitation in choosing a mesh repair [9]. Joint guidelines from the European Hernia Society and the American Hernia Society recommend mesh repair for UH with defects measuring 1 cm or larger [17]. For smaller defects, under 1 cm, the decision to use mesh is left to the discretion of the surgeon and patient. However, a recent retrospective study from Denmark demonstrated a reduction in recurrence rates even for the smallest defects repaired with a mesh technique (3.1%) compared to suture repair (6.7%) [5]. Currently, limited published scientific data are available on the surgical methods used for UHR and their outcomes in Sweden.

This study aimed to investigate the trends and prevalence of suture versus mesh repairs for UHRs in Sweden over time and to assess whether treatment practices have evolved following the publication of recent guidelines. Additionally, the study examined regional variations in repair techniques, different mesh repairs and presents demographic data on the operated population. The hypothesis was that the proportion of mesh-based repairs compared to suture repairs has significantly increased over the study period.

## Methods

### Study Design

This is a nationwide, population-based registry-based cohort study with prospectively collected data from the Swedish Perioperative Registry (SPOR). All primary UHRs conducted on patients aged 18 years and above, operated on between 1 January 2017 and 31 December 2022 and registered in the SPOR, were eligible for the study. Data are presented according to the STROBE guidelines for observational studies [18]. Prior to data extraction, the study protocol received ethical approval from the Swedish Ethical Review Authority (Dnr 2023-00373-01). Oral informed consent is obtained of all patients before inclusion in SPOR. Since this registry study is based solely on anonymized and aggregated data and does not include any identifiable images or individual-level photographs, written informed consent for publication was not required.

### Study Population

**FIGURE 1:**
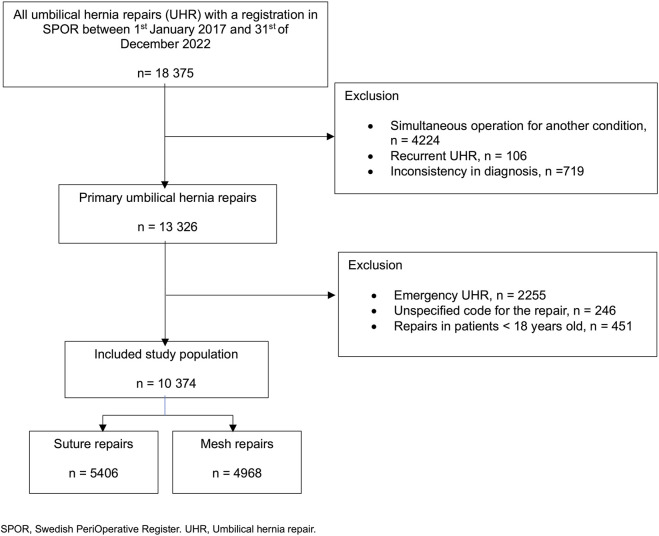
Flowchart of eligible umbilical hernia repairs.

All patients who underwent a primary elective UHR as classified by the Swedish procedural coding system with a registry in SPOR were eligible for inclusion in the study (Figure 1). Excluded UHRs were i) repairs with an unknown method of repair, ii) an UHR registered in SPOR, performed as part of another procedure, and iii) emergency UHRs (Figure 1). In cases where patients had multiple entries for UHR, only the first entry was included. UHR entries with inconsistencies between the diagnosis code and the recorded type of operation were excluded (Figure 1). After applying inclusion and exclusion criteria, a total of 10,374 elective primary umbilical hernia repairs (UHRs) in patients aged 18 years and older were included in the final study population (Figure 1).

### Outcomes

The primary outcome was change over time in the proportion of UHs repaired with mesh versus suture and to assess whether treatment practices have evolved following the publication of 2020 guidelines [17].

The secondary outcomes were demographics of patients undergoing UHR, and to evaluate regional differences in the surgical management of UHs across Sweden during the study period.

### Database

The SPOR was established in 2013 and is a national quality registry initiated by Swedish Association for Anaesthesia and Intensive care [19]. It collects data from the operation planning system in all public hospitals in Sweden. Data are recorded prospectively by the surgical and anesthesia teams. This includes patient demographics, comorbidities, and preoperative risk assessments, along with essential perioperative details including anesthesia methods, type of procedure, intraoperative events, and diagnostic classifications. No surgical outcomes, such as recurrence or complications after surgery are registered in the SPOR. Surgeons are responsible for assigning the appropriate diagnostic and procedural codes for each operation. Over the study period, all publicly operated hospitals in Sweden, except one, submitted data to the SPOR. The coverage rate increased from 85% in the first year of the study period to 99% in the final year [20].

### Variables

All hernia and patient demographics were collected from the SPOR. Demographic variables included patient characteristics of age, sex and the American Society of Anaesthesiologists (ASA) physical status classification system. ASA class was grouped into two categories (1-2 and 3-4) (Table 1). Height and weight data were obtained and body mass index (BMI) in kg/m^2^ was subsequently calculated. According to the Swedish surgical procedure coding system, umbilical hernia repair is classified as either suture repair or mesh repair (Table 1). Mesh repair is further categorised based on the surgical approach—open or laparoscopic (Table 2)—and according to the anatomical placement of the mesh: onlay, interstitial, inlay, sublay, or intraperitoneal onlay mesh (IPOM) (Figure 4). Onlay placement refers to mesh positioned above the aponeurosis. Inlay mesh is defined as being situated within the hernia defect. Sublay mesh placement is defined as an implant positioned in either the retromuscular or preperitoneal plane. IPOM (intraperitoneal onlay mesh) denotes mesh placement within the peritoneal cavity. Interstitial mesh placement lacks a clear definition in the literature and was best considered an indeterminate form of mesh use.

**TABLE 1:** Patient characteristics for the included study population.

Characteristic	Suture repair N = 5,406 (52.1)	Mesh repair N = 4,968 (47.9)	Total N = 10,374	p-value**
Sex	​	​	​	<0.001
Female	2025 (37.5)	1,280 (25.8)	3,305 (31.9)	​
Male	3,375 (62.4)	3,675 (74.0)	7,050 (68.0)	​
Missing	6 (0.1)	13 (0.2)	19 (0.18)	​
Age, years*	​	​	​	<0.001
Female	40 (34, 49)	44 (36, 57)	42 (34, 52)	​
Male	54 (45, 65)	57 (49, 66)	56 (47, 65)	​
Total	49 (39, 61)	55 (45, 64)	52 (41, 63)	​
BMI, kg/m^2^*^	​	​	​	<0.001
Female	24.6 (22.0, 28.3)	28.0 (23.8, 33.2)	25.7 (22.7, 30.2)	​
Male	28.3 (26.0, 30.7)	30.0 (27.5, 33.1)	29.2 (26.8, 32.1)	​
Total	27.2 (24.4, 30.1)	29.7 (26.7, 33.1)	28.4 (25.4, 31.6)	​
ASA class	​	​	​	<0.001
1–2	4,635 (85.7)	3,983 (80.2)	8,618 (83.1)	​
3–4	588 (10.9)	848 (17.1)	1,436 (13.8)	​
Missing	183 (3.4)	137 (2.8)	320 (3.1)	​
Healthcare region	​	​	​	<0.001
Central healthcare region	1,505 (27.8)	1,417 (28.5)	2,922 (28.2)	​
Northern healthcare region	628 (11.6)	553 (11.1)	1,181 (11.4)	​
Southern healthcare region	740 (13.7)	550 (11.1)	1,290 (12.4)	​
Stockholm and gotland Healthcare region	834 (15.4)	710 (14.3)	1,544 (14.9)	​
South-eastern healthcare Region	511 (9.5)	487 (9.8)	998 (9.6)	​
Western healthcare region	1,188 (22.0)	1,251 (25.2)	2,439 (23.5)	​
Annual cohorts***	​	​	​	0.063
First year (2017)	803 (53.8)	690 (46.2)	1,493	​
Last year (2022)	1,069 (50.6)	1,044 (49.4)	2,113	​

Data is presented in numbers (n) and percentage (%) within parentheses if not indicated otherwise. *Median and IQR (25–75 percentile) within parentheses. ASA, class; American Society of Anaesthesiologists classification. BMI; Body Mass Index. ^Missing data on BMI, n = 1986 UHR. **p-values; Continuous variables were analysed using the Wilcoxon rank sum test, and categorical variables were analysed using Pearson’s chi-squared test. *** Percentages calculated on yearly cohort.

**TABLE 2:** Patient characteristics by open or laparoscopic mesh repair for the study population.

Characteristic	Open repair N = 4,265 (85.8)	Laparoscopic repair N = 703 (14.2)	p-value**
Sex	​	​	<0.001
Female	1,048 (24.6)	232 (33.0)	​
Male	3,206 (75.1)	469 (66.7)	​
Missing	11 (0.3)	2 (0.3)	​
Age, years*	​	​	<0.001
Female	44 (36, 56)	47 (38, 58)	​
Male	57 (49, 66)	57 (50, 65)	​
Total	55 (45, 65)	55 (46, 63)	​
BMI, kg/m^2^*^	​	​	<0.001
Female	27.3 (23.6, 32.3)	30.9 (24.7, 35.3)	​
Male	29.7 (27.4, 32.7)	32.4 (28.7, 35.1)	​
Total	29.4 (26.5, 32.6)	31.9 (27.8, 35.3)	​
ASA class	​	​	0.040
1–2	3,439 (80.6)	544 (77.4)	​
3–4	717 (16.8)	131 (18.6)	​
Missing	109 (2.6)	28 (4.0)	​
Healthcare region	​	​	<0.001
Central healthcare region	1,181 (27.7)	236 (33.6)	​
Northern healthcare region	479 (11.2)	74 (10.5)	​
Southern healthcare region	498 (11.7)	52 (7.4)	​
Stockholm and gotland Healthcare region	478 (11.2)	232 (33.0)	​
South-eastern healthcare region	469 (11.0)	18 (2.6)	​
Western healthcare region	1,160 (27.2)	91 (12.9)	​
Annual cohorts***	​	​	<0.001
First year (2017)	614 (89.0)	76 (11.0)	​
Last year (2022)	826 (79.1)	218 (20.9)	​

Data is presented in numbers (n) and percentage (%) within parentheses if not indicated otherwise. *Median and IQR (25–75 percentile) within parentheses. ASA, class; American Society of Anaesthesiologists classification. BMI; Body Mass Index. ^Missing data on BMI, n = 720. Laparoscopic repairs included 5 repairs conducted with robotic approach. **p-values; Continuous variables were analysed using the Wilcoxon rank sum test, and categorical variables were analysed using Pearson’s chi-squared test. *** Percentages calculated on yearly cohort.

Healthcare units were classified into six geographically predefined and autonomous regions in Sweden, as determined by the Swedish National Board of Health and Welfare (Table 3). Each region independently organises and delivers specialised surgical care, and operates under a national framework intended to ensure comparable prerequisites for optimal and equitable surgical care.

**TABLE 3:** Patient characteristics by healthcare region for suture and mesh repairs.

Health care region	Type of repair	Sex		Age, years*	BMI, kg/m^2^*	ASA class	
		Female	Male			1–2	3–4
Central healthcare region	Suture repair, N = 1,505 (51.5)	555 (62.6)	948 (46.8)	49 (39–61)	27.5 (24.5–30.4)	1,331 (53.6)	126 (33.8)
	Mesh repair, N = 1,417 (48.5)	332 (37.4)	1,078 (53.2)	55 (45–65)	29.8 (27.0–33.6)	1,150 (46.4)	247 (66.2)
Northern healthcare region	Suture repair, N = 628 (53.2)	227 (65.2)	401 (48.2)	48 (37–60)	27.2 (24.6–29.8)	503 (54.2)	71 (42.5)
	Mesh repair, N = 553 (46.8)	121 (34.8)	431 (51.8)	55 (46–63)	29.9 (27.2–33.2)	425 (45.8)	96 (57.5)
Southern healthcare region	Suture repair, N = 740 (57.4)	251 (64.0)	488 (54.4)	51 (39–63)	27.8 (25.1–30.4)	640 (58.7)	98 (49.7)
	Mesh repair, N = 550 (42.6)	141 (36.0)	409 (45.6)	55 (46–65)	30.1 (27.5–33.6)	451 (41.3)	99 (50.3)
Stockholm and gotland healthcare region	Suture repair, N = 834 (54.0)	321 (61.0)	510 (50.3)	50 (41–63)	26.7 (23.9–29.8)	663 (57.4)	121 (45.1)
	Mesh repair, N = 710 (46.0)	205 (39.0)	504 (49.7)	55 (45–65)	29.5 (26.6–32.8)	493 (42.6)	147 (54.9)
South-eastern healthcare region	Suture repair, N = 511 (51.2)	175 (57.6)	336 (48.4)	50 (40–63)	27.7 (25.7–30.6)	438 (51.6)	66 (48.5)
	Mesh repair, N = 487 (48.8)	129 (42.4)	358 (51.6)	54 (45–65)	29.2 (26.1–31.2)	411 (48.4)	70 (51.5)
Western healthcare region	Suture repair, N = 1,188 (48.7)	496 (58.5)	692 (43.6)	48 (38–60)	26.8 (23.9–29.7)	1,060 (50.2)	106 (35.9)
	Mesh repair, N = 1,251 (51.3)	352 (41.5)	895 (56.4)	54 (45–64)	29.3 (26.2–32.8)	1,053 (49.8)	189 (64.1)

Numbers (n), percentage (%) within parentheses if not indicated otherwise. *Median, IQR (25–75 percentile) within parentheses.

ASA, class: American Society of Anaesthesiologists classification. BMI: Body Mass Index. ^Missing data: Sex, n = 19, BMI, n = 1986, ASA, class, n = 320.

### Statistical Analysis

**FIGURE 2:**
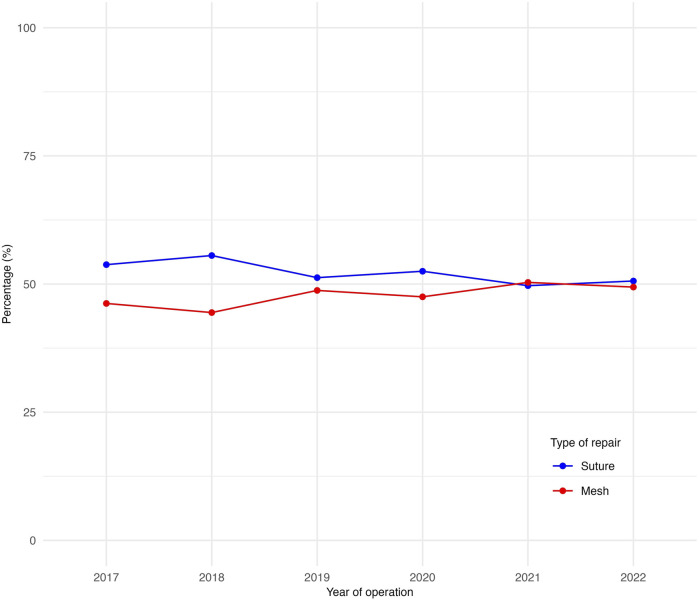
Proportions of suture versus mesh repairs over time during the study period.

**FIGURE 3:**
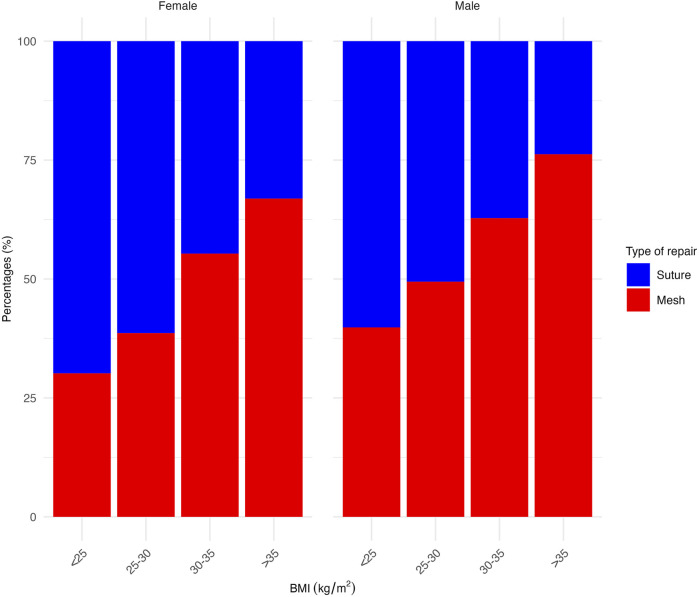
Proportion of mesh versus suture repair across different BMI groups, separated by sex.

Data were analysed according to a prewritten study protocol. Missing data patterns were explored descriptively. BMI missingness was assessed in relation to patient characteristics and surgical technique. Ad hoc analyses were conducted to explore the relationship between BMI, sex, and mesh use (Figure 3). Pearsons’s chi-square test was used to analyse categorical variables and to compare differences in mesh use between the first and last years of the study period (Figure 2; Tables 1,2). The Mann-Whitney U test was used to compare continuous variables (Tables 1,2). Categorical variables were described with numbers and percentages (Tables 1–3). Continuous variables were presented with medians and interquartile ranges (Tables 1–3). P-values are provided to indicate statistical comparisons (Tables 1,2). All data analyses were performed using RStudio (Version 2023.12.1 + 402).

## Results

Between 2017 and 2022, a total of 18,375 primary UHRs were registered in the SPOR (Figure 1). After applying the exclusion criteria, 10,374 patients were included in the final study population, consisting of primary elective UHRs. Conducted on patients aged 18 years and older (Figure 1).

### Patient Characteristics

Out of 10,374 UHRs, 5,406 (52.1%) patients received a suture repair, while 4,968 (47.9%) underwent a mesh repair (Table 1). 68.0% of the cohort were men and mesh repairs were significantly more frequently performed on men (52.1%) compared to women (38.7%), *p* < 0.001 (Table 1). The median age of the study population was 56 years for men and 42 years for women. In both the mesh repair and suture repair groups, men had a higher median age compared to women. The median BMI was higher in men (29.2 kg/m^2^) compared to women (25.7 kg/m^2^). Men had a significantly higher BMI in both the suture repair group and in the mesh repair group compared to women. An ASA classification of 1–2 was observed in 83.1% of all UHRs, with a similar distribution across both surgical technique groups (Table 1). Data on BMI were missing in 23.4% of cases in the suture repair group and 14.5% in the mesh repair group. BMI missingness did not differ by sex, age or ASA class. The Central healthcare region performed the highest number of UHRs (28.2%) over the study period (Table 1).

### Suture Versus Mesh Repair

A small increase was observed in the proportion of mesh repairs when comparing the UHRs of the first (46.2%) and last years (49.4%) of the study period (*p* = 0.063) (Figure 2). Patients undergoing mesh repair were significantly older, with a median age of 55 years compared to 49 years in the suture repair group (p < 0.001). They also had a higher proportion of ASA classification 3–4 (17.1% vs. 10.9%, p < 0.001) and a greater median BMI (29.7 kg/m^2^) than those undergoing suture repair (27.2 kg/m^2^) (p < 0.001) (Table 1). The proportion of mesh repairs was greater for patients with a higher BMI across both sexes (Figure 3). This was also demonstrated in an exploratory multivariable analysis where BMI emerged as the strongest predictor of mesh use (estimates not presented).

### Open and Laparoscopic Mesh Repairs

Of the mesh repairs, 4,265 (85.8%) were performed with an open technique and 703 (14.2%) laparoscopically (Table 2). The median BMI was significantly lower in patients undergoing open mesh repair (29.4 kg/m^2^) compared to those who had a laparoscopic repair (31.9 kg/m^2^) (*p* < 0.001). Median age was identical between the groups for men (57 years, p = 0.961), while women undergoing open mesh repair were younger than those undergoing laparoscopic repair (median age 44 years vs. 47 years, *p* = 0.027). Additionally, a significantly lower proportion of laparoscopic mesh repairs for men compared to for women (12.8% vs. 18.1%, *p* < 0.001) was noticed. Stockholm and Gotland healthcare regions had the highest proportion of laparoscopic mesh repairs (Table 2). The laparoscopic repairs had a significant increase over the study periods comparing first versus last year (11.0%–20.9%, *p* < 0.001) (Table 2). In a sensitivity analysis excluding one hospital with late registry entry and high laparoscopic use, the significant difference was no longer observed (11.5% in 2022, *p* = 0.828). Baseline characteristics were comparable between this hospital and the overall cohort. A laparoscopic robotic approach was used in five repairs (Table 2). Of these, two were suture repairs, two involved sublay mesh repair, and one involved an IPOM repair. The median age was 69 years, and the median BMI was 30.7 kg/m^2^. All patients were classified as ASA 1–2.

### Different Mesh Repairs

**FIGURE 4:**
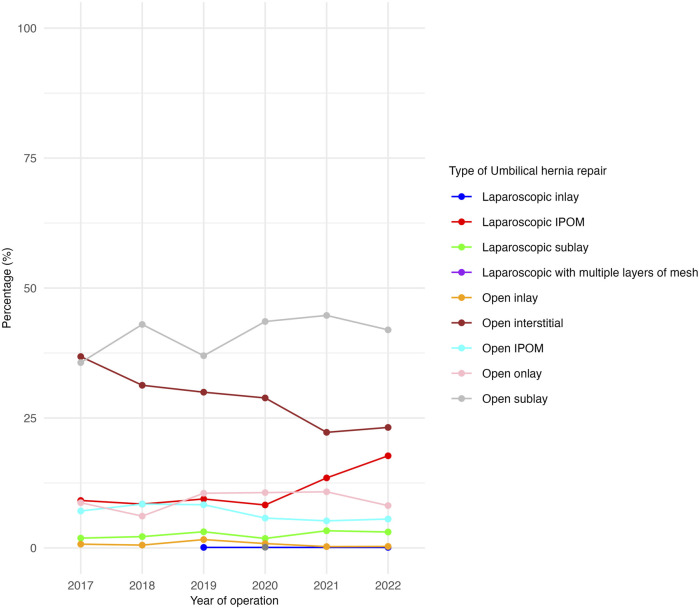
Proportion of umbilical hernia repairs by type of mesh technique during the study period.

Open sublay mesh repairs remained the dominant type of UHR throughout the study period, increasing from 36% to 42% (Figure 4). In contrast, open interstitial repair decreased from 37% to 23% (Figure 4). Laparoscopic IPOM showed an increase from 8% to 18%, primarily during the last two years, while open IPOM remained relatively stable, changing from 7% to 6% (Figure 4).

### Healthcare Regions

Differences between healthcare regions are presented in Table 3. Notably, in all regions, men had a higher proportion of mesh repairs. Additionally, BMI and age were consistently higher among patients undergoing mesh repairs compared to suture repairs. A significant increase in mesh repairs during the study periods first year versus final year was observed only in the Southern healthcare region, showing a significant rise from 25.0% to 56.1%, *p* < 0.001 (not presented in Table 3).

## Discussion

This large nationwide registry-based cohort study gives an insight into trends, prevalence, and patient demographics of different surgical methods of repairs for UH in Sweden. Findings indicated that mesh was used in fewer than half of all primary UHRs in Sweden, with no clear trend of increasing use during the study period. Men underwent more repairs and were more likely to receive a mesh. Mesh was more commonly used in patients with a higher BMI. Only one healthcare region saw a significant increase in mesh use over the study period.

### Trends in Umbilical Hernia Surgery

Recent years have brought international guidelines [17] and substantial evidence demonstrating a significant reduction in recurrence rates for mesh repair in primary umbilical hernia compared to suture repair [3, 5, 7, 12]. This highlights the importance of increasing the proportion of mesh repairs to improve patient outcomes and reduce the burden of recurrence. Even the smallest umbilical hernias, under 1 cm, appear to have an increased risk of recurrence when repaired with sutures alone [5]. However, despite the growing body of evidence supporting mesh use over suture repairs, the present study does not demonstrate an increase in the proportion of mesh repairs. In England, mesh has been reported to be used at a similar rate for primary UHRs (50%) [21] to the one observed in the present study. A recent retrospective study from the United States demonstrated a higher proportion of mesh use, 67% for men and 60% for women, and with a similar sex distribution of operated UH patients [22] as in the present study. The sex distribution in umbilical hernia repair has shifted from female predominance in the early 2000s [14] to male dominance in recent years [2, 3, 5]. This male predominance was also observed in this study. Whether this reflects a true change in incidence, potentially related to rising obesity and comorbidity among men, or is driven by evolving, sex-specific treatment thresholds and broader sociocultural factors remains unclear.

Concerns have been raised regarding the increased risk of surgical site occurrences with mesh [9, 12, 23]. This could influence the low number of repairs performed with mesh, especially when defects are smaller. A lack of familiarity with guidelines and current evidence may also contribute to variations in UH treatment. While all surgeons are expected to be proficient in UHR due to its common nature, their knowledge of the latest recommendations and adherence to guidelines may vary.

Findings from studies such as this highlight the ongoing challenge of guideline adherence of surgical managment of umbilical hernias and emphasize the importance of continuous education. Additionally, presentations and discussions at conferences are vital for raising awareness and promoting the active pursuit of the most current evidence. Further research incorporating hernia-specific variables, such as defect size, is necessary to determine whether current clinical practices are already aligned with the guidelines or if there remains a gap in their implementation into routine care.

### Patient Characteristics

Although the hernia size data were not collected in this study due to limitations in the SPOR, previous research has demonstrated that an increasing BMI is associated with larger hernia defects [22]. In the present study, the proportion of mesh repairs increased for UHRs performed on patients with a higher BMI in both sexes. However it remains unclear whether the increased use of mesh was driven by larger hernia defects or by a preference among surgeons to use mesh more frequently in patients with higher BMI due to concerns of a potentially increased risk of recurrence [24]. Patients undergoing mesh repair were also older and had a higher ASA class compared to those receiving suture repair. Probably due to the fact that mesh is often preferred for more complex cases, larger hernias, or in patients with a higher BMI, which are more common in an older population. However, the underlying reasons for these associations could not be explored in greater details within the scope of the available data.

### Surgical Techniques

Sublay repair was the predominant mesh repair technique. Current guidelines advocate for preperitoneal placement, which is considered to be a retrorectus positioning of the mesh in this classification. According to the registry, interstitial mesh placement was the second most frequently reported technique. However, this anatomical plane is not clearly defined in umbilical hernia surgery, and the majority of these registrations are more likely to be considered as representing an unspecified open mesh approach.

There was a significant increase in laparoscopic mesh repairs between the first and last years of the study period. This despite the debate regarding IPOM and its associated risks for intraoperative and long-term complications [25, 26]. This increase was considered driven by one high-volume center performing many IPOM repairs, reporting data only in the last 2 years. Patient characteristics were similar to the overall cohort, suggesting the higher laparoscopy use likely reflects a center-specific technical preference. Guidelines recommend a laparoscopic approach for defects larger than 4 cm or in repairs with a high risk of surgical site infection [17]. In this study, the BMI for patients undergoing laparoscopic repairs was significantly higher compared to the BMI for patients undergoing open mesh repairs. A higher BMI has been shown to increase the risk of surgical site infections in open ventral hernia repairs [27]. This may explain why patients with UH and a higher BMI are more likely to be selected for a laparoscopic repair.

### Healthcare Regions

Furthermore, a comparison across different healthcare regions revealed similar patterns in the choice of mesh repair versus suture repair, with consistent associations observed for BMI, age and sex. The observed increase in the proportion of mesh repairs from the first to the last year of the study in the southern region may be partly explained by a decline in suture repairs at some hospitals, while mesh repairs showed a modest increase in others within the region. No systematic differences in treatment options and overall service provision are expected between the regions for UH repairs. In 2019, a hospital with a relatively high proportion of mesh repairs was incorporated into SPOR, which may also have contributed to the significant trend in the southern region. This suggests that observed changes are more likely driven by local practice patterns at individual surgical units rather than regional or national policy shifts.

This study is, to our knowledge, the first to present prevalence and trends of different surgical methods for primary UHRs in Sweden alongside their patient demographics within a large nationwide cohort. The use of prospectively collected data from a highly validated national registry, covering nearly all hospitals in Sweden, ensures an accurate depiction of trends and patient demographics. The comprehensive nature of the data allows for a robust analysis of clinical practices across various patient subgroups. Furthermore, the large sample size provides sufficient statistical power to detect differences and trends over time.

However, this study has some limitations. SPOR is not a dedicated hernia database and has limitations regarding hernia-specific data points and outcomes. The most significant limitation was the lack of information on the UH defect size. This variable was not recorded in the SPOR. Moreover, although the majority of public surgical units report to the SPOR, smaller and private surgical units are known to omit reporting. This limitation means that the cohort was not entirely representative of the national population. It is possible that private healthcare providers more commonly operate on smaller defects using an open technique, which is often associated with outpatient surgery. Mesh use and laparoscopic techniques more frequently require hospital admission and more advanced anesthetic management, which may not always be available at smaller private units. BMI missingness was considered potentially missing not at random, limiting the feasibility of multivariable analyses including BMI. The higher rate of missing BMI in suture repairs may reflect smaller, less complex hernias, for which BMI is less consistently recorded, and is therefore unlikely to substantially affect the descriptive conclusions of this study. Additionally, no data on surgical outcomes, including recurrences or other complications following surgery, are registered in the SPOR, which would have enabled an investigation into these events in respect to this patient cohort. No databases in Sweden currently record these variables with a high coverage rate.

In conclusion, umbilical hernias in Sweden are still predominantly repaired with suture repair. Mesh was used more frequently among men, obese patients, and those with greater co-morbidities. No clear trend indicating an increase in mesh use for UHR was observed during the study period. These findings suggest that mesh repairs may not have substantially influenced UHR practices in Sweden over the study period. Future studies incorporating UH defect size could further enhance the understanding of UHRs and contribute to the ongoing recommendations on selecting the most appropriate surgical technique.

## Data Availability

The raw data supporting the conclusions of this article will be made available by the authors, without undue reservation.
